# The role of transketolase and octulose in the resurrection plant *Craterostigma plantagineum*

**DOI:** 10.1093/jxb/erw174

**Published:** 2016-04-29

**Authors:** Qingwei Zhang, Thomas Vitus Linnemann, Lukas Schreiber, Dorothea Bartels

**Affiliations:** ^1^Institute of Molecular Physiology and Biotechnology of Plants (IMBIO), University of Bonn, Kirschallee 1, 53115 Bonn, Germany; ^2^Institute of Cellular and Molecular Botany, Department of Ecophysiology, University of Bonn, Kirschallee 1, 53115 Bonn, Germany

**Keywords:** Octulose, photosynthesis, resurrection plants, sugar transport, transketolase.

## Abstract

Phylogenetic analysis revealed that *Craterostigma plantagineum* has two transketolase genes (*transketolase 7* and *10*) which are separated from the other transketolase genes including *transketolase 3* from *C. plantagineum*. We obtained recombinant transketolase 3, 7, and 10 of *C. plantagineum* and showed that transketolase 7 and 10 of *C. plantagineum*, but not transketolase 3, catalyse the formation of octulose-8-phosphate *in vitro*. Transketolase 7 and 10 of *C. plantagineum* performed the exchange reaction that produces octulose-8-phosphate using glucose-6-phosphate and fructose-6-phosphate as substrates. Octulose is localized in the cytosol and phloem exudate analysis showed that octulose was the dominant sugar exported from the leaves to the roots.

## Highlight

Transketolase 7 and 10 of *Craterostigma plantagineum* participate in the synthesis of octulose phosphate in an alternative Calvin cycle. Octulose is the main transport sugar in fully hydrated plants.

## Introduction

d-Glycero-d-ido-octulose (octulose) is a rare monosaccharide which accumulates in the desiccation-tolerant plant *Craterostigma plantagineum* Hochst. The conversion between octulose and sucrose occurs when *C. plantagineum* undergoes dehydration and subsequent rehydration (Bianchi *et al.*, 1991). Considerable research has revealed that sucrose has a fundamental function in regulating osmotic potential and protecting membranes as well as macro-molecules in resurrection plants (Hoekstra *et al.*, 1997; Vicré *et al.*, 2004; Peters *et al.*, 2007; Dinakar and Bartels, 2013). Some efforts have been made to explain how sucrose is produced in *C. plantagineum* (Ingram *et al.*, 1997; Kleines *et al.*, 1999). In contrast to sucrose metabolism, the generation and physiological function of free octulose is still a mystery.

Octulose is an eight carbon monosaccharide, its mono-phosphate octulose-8-phosphate is an intermediate in the pentose phosphate pathway and may be synthesized via a novel alternative photosynthesis pathway (Flanigan *et al.*, 2006; Williams and MacLeod, 2006). Transketolase acts as a key enzyme in the pentose phosphate pathway and, in photosynthesis, it catalyses the formation of various sugar phosphates (Krüger and von Schaewen, 2003). It has been suggested to participate in octulose metabolism in *C. plantagineum*. Three isoforms of transketolase (tkt3, tkt7, and tkt 10) have been characterized at the molecular level in *C. plantagineum* (Bernacchia *et al.*, 1995). Transketolase extracted from rehydrated leaves of *C. plantagineum* catalysed the formation of octulose-8-phosphate using glucose-6-phosphate and β-hydroxypyruvate as substrates (Willige *et al.*, 2009). Similarly, Williams and MacLeod (2006) obtained octulose-8-phosphate in extracts from spinach through the exchange reaction with glucose-6-phosphate and fructose-6-phosphate as substrates. They proposed that the exchange reaction catalysed by transketolase is part of an alternative Calvin cycle (also called the alternative scheme). Although there is still a dispute about the exchange reaction, Clasquin *et al.* (2011) provided new evidence for this alternative scheme. They showed that d-glycero-d-altro-octulose-1,8-*bis*phosphate might be synthesized in yeast by the aldol addition of dihydroxyacetone phosphate and ribose-5-phosphate which was catalysed by the glycolytic enzyme fructose *bis*phosphate aldolase. This is consistent with another part of the alternative scheme proposed by Flanigan *et al.* (2006). Therefore, it is necessary to examine whether the exchange reaction is performed by transketolase isolated from *C. plantagineum*. Storage and transport are important components of sugar metabolism which are involved in carbon allocation in plants. The appearance of octulose in the roots of *C. plantagineum* (Norwood *et al.*, 2003) raises the question, could octulose be transported?

Transketolase protein was extracted from *C. plantagineum* leaves and the recombinant *C. plantagineum* transketolases 3, 7, and 10 were obtained from the corresponding over-expression construct. Proteins were used to test the exchange reaction. GC/MS analysis demonstrated that transketolases 7 and 10 can perform the exchange reaction and octulose was synthesized. Octulose is accumulated in the cytosol, acting as an excellent antioxidant (Zhang and Bartels, 2016). Octulose is also exported from the leaves to the roots in *C. plantagineum*.

## Materials and methods

### Plant material

*C. plantagineum* plants were grown as previously described by Bartels *et al.* (1990).

### Gene cloning and protein purification

The cDNA fragments encoding the *C. plantagineum* genes *TKT3*, *TKT7*, and *TKT10* were amplified by PCR and cloned into the PJET1.2 vector according to the manual of molecular cloning (Sambrook *et al.*, 2001), the guide to the CloneJET PCR cloning kit (Thermo scientific, #k1231), and the Thermo scientific protocol of DNA digestion and ligation. Detailed information on the gene sequences of *TKT3*, *TKT7*, and *TKT10* of *C. plantagineum* is given by Bernacchia *et al.* (1995). Enzyme-digested fragments were ligated to the digested pET28a+ vector and transformed into the *Escherichia coli* expression strain BL21 (DE3). All of the primers used were designed by software Oligo 7 and their sequences are listed in Supplementary Table S1 at JXB online. Protein expression and purification were conducted according to Kirch and Röhrig (2010). Purified proteins were dissolved in the elution buffer: 50mM HEPES/NaOH (pH 7.4), 300mM NaCl, 250mM imidazole, 10% (v/v) glycerol, 0.1% (v/v) Triton X-100, and 1.5mM β-mercaptoethanol. The empty vector pET 28a+ was used as the control in the transformation and protein purification from *E. coli* BL21 (DE3) cells. Protein concentrations were determined according to Bradford (1976) using the Bio-Rad kit (Bio-Rad Laboratories, Inc. USA).

### Transketolase extraction from plant tissue and protein analysis

Transketolase from plant leaves was purified according to Bernacchia *et al.* (1995). The pellet that was obtained after 50–70% (w/v) (NH_4_)_2_SO_4_ precipitation was dissolved in 500 µl buffer A (50mM TRIS–HCI, pH 7.5, 10% (v/v) glycerol, 10mM MgCl_2_) and used for enzymatic assay.

Sodium dodecyl sulphate polyacrylamide gel electrophoresis (SDS–PAGE) of the proteins was conducted according to Laemmli (1970). Protein Western blot analysis was performed as described by Bartels *et al.* (1991) and the transketolase antiserum was prepared by Bernacchia *et al.* (1995).

### Enzymatic assays and product dephosphorylation

The enzymatic reactions were performed as described by Willige *et al.* (2009): 25 µg purified protein, 58mM glycylglycine (pH 7.7), 0.01% (w/v) Na-azide, 0.002% thiamine pyrophosphate, 15mM MgCl_2_, 5.3mM acceptor substrate (ribose-5-phosphate or glucose-6-phosphate), and 16mM donor substrate (β-hydroxypyruvate or fructose-6-phosphate).

After the catalysing reaction, sugar phosphates were dephosphorylated according to Willige *et al.* (2009). The dephosphorylated products were purified by passing through a column containing ion-exchange bed resin AG 501-X8(D) (BIO-RAD). The flow-through fractions were used for GC/MS analysis.

### Carbohydrate extraction and sugar analysis by GC/MS

Sugars were extracted from plant tissues as described by Willige *et al.* (2009). Freeze-dried plant material was ground to a fine powder and extracted twice with 80% (v/v) methanol (3ml g^–1^) at 4 °C. The homogenates were cleared by centrifugation for 5min at 5 000 *g* and 4 °C. The methanol was evaporated to dryness under reduced pressure at 25 °C. The sediment was taken up in water and washed three times with chloroform to remove lipophilic substances. The aqueous phase was centrifuged for 30min at 10 000 *g* and 4 °C to remove any particles. A cation (Dowex 50 WX8) and an anion (Dowex IX8) exchange resin were added to the aqueous phase (5g resin per 100ml) to remove organic acids, amino acids or other charged molecules. After stirring for 1h the aqueous phase was transferred into a reaction vial for GC analysis.

The extracted sugar fractions were further separated and identified by coupled gas chromatography (GC)/flame ionization detection (GC/FID) and coupled gas chromatography/mass spectrometry (GC/MS). Ten µl of extract, prepared as described above, and 10 µg of xylitol (used as the internal standard) were dried at 60 °C under N_2_ gas. 30 µl pyridine and 30 µl N,O-Bis(trimethylsilyl)-trifluoracetamide (BSTFA) were then added and the sample was diluted with 50 µl chloroform to reach a mass between 1ng and 20ng. The samples were heated for 40min at 70 °C. The trimethylsilyl (TMS) sugar derivatives were separated on a DB1 column (J&W Scientific, Folsom, CA, USA). Qualitative GC/MS analysis was carried out with a gas chromatograph 7890B, detector 5977A MS Detection HP (Agilent Technologies, Böblingen, Germany); quantitative analysis was carried out with GC/FID (5890Series II Plus, HP, Agilent Technologies) (Willige *et al.*, 2009). One µl of each sample was injected and the H_2_ flow was set to 37 kpa. The initial temperature was 65 °C for 3min, after which the temperature was raised at a rate of 8 °C min^–1^ to a temperature of 240 °C, after which the rate was increased by 12 °C min^–1^ to a final temperature of 310 °C for 35min. Data analysis was performed with GC ChemStation [Rev.B.03.02(341), Agilent Technologies, Böblingen, Germany].

### Chloroplast isolation

Chloroplasts of *C. plantagineum* were isolated according to Rowan and Bendich (2011). Plant leaves were rinsed briefly with 70% (v/v) ethanol, followed by distilled water. Four grams of *C. plantagineum* leaves were mixed into 100ml XPI solution (50mM HEPES pH 7.5, 330mM sorbitol, 5mM ascorbic acid, 1mM MgCl_2_, 1mM MnCl_2_, and 2mM EDTA) and homogenated on ice using a blender. The homogenate was filtered through three layers of Miracloth. The filtered liquid was centrifuged at 4 °C at 3 000 *g* for 5min. The pellet was suspended in 5ml XPI solution and layered on to the prepared 30/70% Percoll gradient (70% Percoll at the bottom, 30% Percoll on top). The mix was centrifuged at 4 °C at 12 000 *g* for 10min using a swing-out rotor. The purified chloroplasts were collected from the band at the interface between the 30% and 70% Percoll layers using glass Pasteur pipettes. The chloroplasts were washed twice with 10 vols of XPI solution and centrifuged at 12 000 *g* for 20s. Finally, the isolated chloroplasts were freeze-dried and kept at –70 °C.

### Thin layer chromatography (TLC) and octulose purification

Sugars were separated by TLC silica gel 60 (CAS 105553, Merck Millipore) using *n*-butanol:H_2_O:*n*-propanol (9:1:0.5 by vol.) as the developing solution for about 2h. The plate was then dried at 37 °C and the sugars were detected by spraying the plates with a mixture of EtOH:H_2_SO_4_:HAc:anisaldehyde (90:5:1:5 by vol.) and heating to 100 °C for 5min (Kutzer, 2004).

The area containing the sugars was excised from the silica gel and saturated in ethanol overnight. After centrifuging at maximum speed for 10min and filtration through a 40 µm filter membrane, the ethanol solution was evaporated to dryness under reduced pressure at 25 °C. Finally, the syrup was weighed and dissolved in water for GC/MS analysis.

### Phloem exudate analysis

The analysis was performed according to Wingenter *et al.* (2010). At the end of the illumination, the petiole ends of mature leaves from 6-week-old plants were cut under water and then transferred quickly into reaction tubes containing 100 µl of 15mM EDTA solution (pH 7.25). The sugar export experiment was done in a water-saturated atmosphere overnight. The solution was then heated to 95 °C for 3min in the reaction tubes before being cooled down for GC analysis (5mM CaCl_2_ inthe reaction tubes was used as a control). To increase the sugar concentration for the GC analysis, the leaf exudate solutions were condensed to about 20 µl by evaporating under reduced pressure at 25 °C. The sugar content is presented as a percentage of the total sugars.

### Phylogenetic analysis of transketolase genes

Transketolase sequences were retrieved by Blast from the National Center for Biotechnology Information (www.ncbi.nlm.nih.gov/) and obtained from the RNA sequencing of *Lindernia brevidens* and *Lindernia subracemosa* (unpublished data). The phylogenetic tree was generated from aligned sequences of predicted proteins from 51 plant transketolase genes by Maximum Likelihood Analysis using 2 000 bootstrap predictions and the 50% majority rule in MEGA6 (Hall, 2013).

## Results

### Molecular phylogeny of plant transketolases

**Fig. 1.**
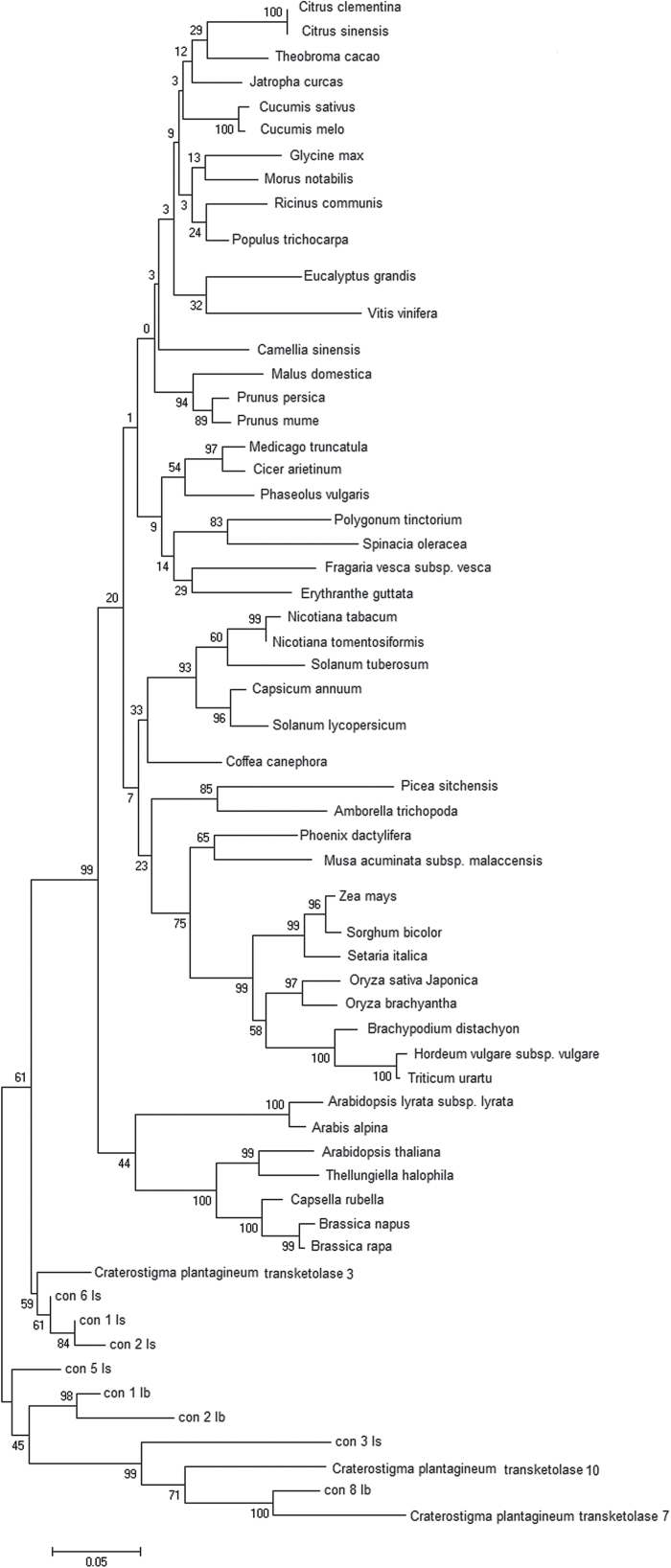
Phylogenetic tree of plant transketolases constructed by Mega 6 software using amino acid sequences obtained from NCBI databases and the transcriptomes of *Lindernia brevidens and Lindernia subracemosa*. The homologues of transketolase genes from *L. brevidens* are con 1 lb, con2 lb, and con 8 lb and from *L. subracemosa* are con 1 ls, con 2 ls, con 3 ls, con 5 ls, and con 6 ls.

Phylogeny analysis of the transketolase genes demonstrates that TKT7 and TKT10 are closely related in *C. plantagineum* and have diverged from other transketolase genes of desiccation-sensitive species while TKT3 shares a higher identity with other plant species (Fig. 1). Using RNA sequencing, we have found eight possible homologous genes for TKT3, TKT7, and TKT10 of *C. plantagineum* in another two octulose-producing plants *L. brevidens* and *L. subracemosa* (Phillips *et al.*, 2008). In the phylogenetic tree, TKT7 and TKT10 and their five homologues from *L*. *brevidens* and *L. subracemosa* (con 8 lb, con 3 ls, con 2 lb, con 1 lb, and con 5 ls) form a separate branch. Three homologues from *L*. *brevidens* and *L. subracemosa* (labelled as con 6 LS, con 1 LS, and con 2 LS) show highly similar evolutionary characteristics with TKT3 in another branch. The branch with *C. plantagineum* TKT3 appears closer to the big group that is composed of transketolases from another 48 angiosperm species. This shows the specificity of the TKT7 and TKT10 genes in octulose-producing plants in evolution.

### *In vitro* synthesis of octulose

**Fig. 2.**
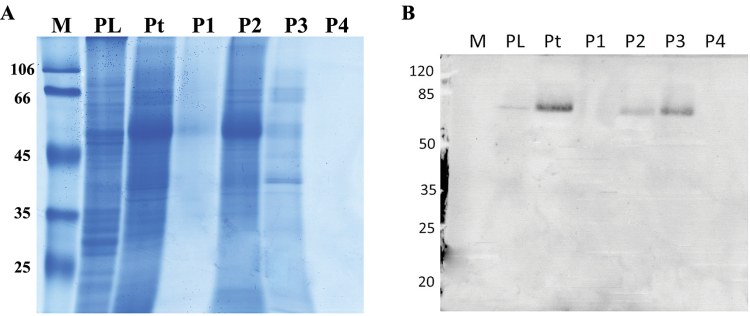
SDS-PAGE (a) and immuno blotting (b) of proteins extracted from leaves of *C. plantagineum*. Note: M, protein size markers (given in kDa); PL, proteins not soluble in extraction buffer; Pt, total proteins extracted by the extraction buffer; P1, proteins precipitated by 0–25% (w/v) (NH_4_)_2_SO_4_; P2, proteins precipitated by 25–50% (w/v) (NH_4_)_2_SO_4_; P3, proteins precipitated by 50–70% (w/v) (NH_4_)_2_SO_4_; P4, proteins remaining in extraction buffer after precipitation by 50–70% (w/v) (NH_4_)_2_SO_4_; all pellets were dissolved in buffer A of the same volume as used in the extraction. Samples were loaded with SDS buffer on the gel.

**Fig. 3.**
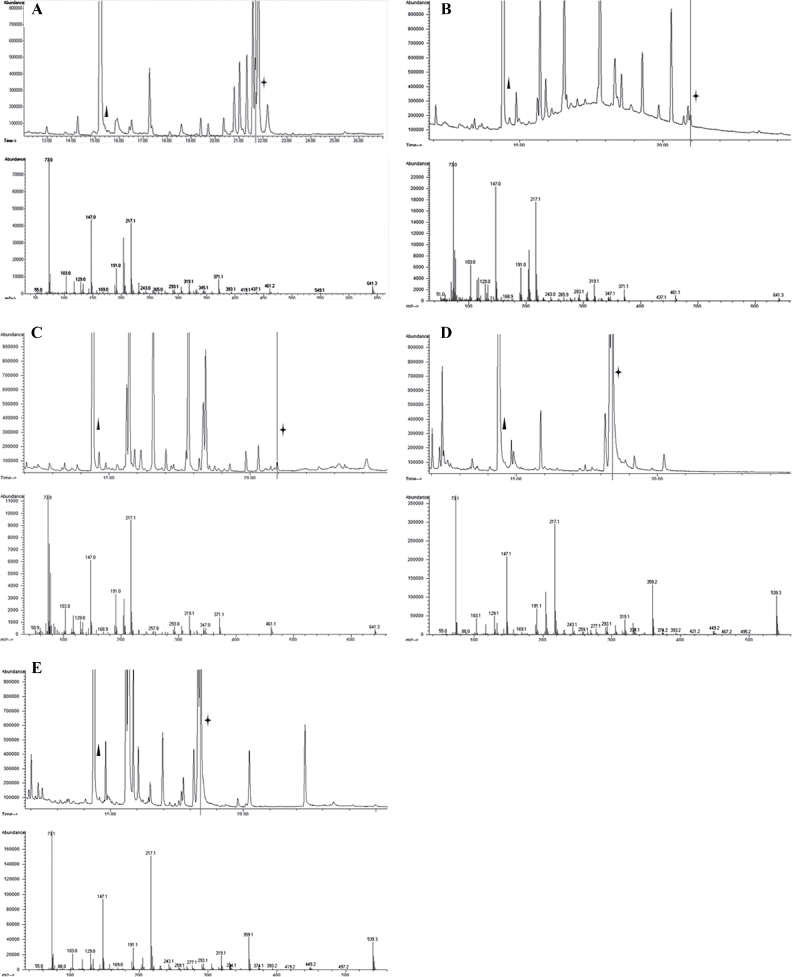
(A) The chromatogram of the trimethylsilyl (TMS) derivatives of octulose purified from *C. plantagineum* leaves by TLC (upper part) and the mass spectra of the TMS derivative of octulose (lower part). The peak corresponding to the internal standard xylitol is labelled by a triangle and that of octulose is labelled by an asterisk. (B) The chromatogram of the TMS derivatives of the dephosphorylated reaction products with glucose-6-phosphate as acceptor substrate and β-hydroxypyruvate as donor (upper part) and the mass spectra of the TMS derivative of octulose (lower part). The peak corresponding to the internal standard xylitol is labelled by a triangle and that of octulose is labelled by an asterisk. The reaction contains 25 µg purified protein, 58mM glycylglycine (pH 7.7), 0.01% (w/v) Na-azide, 0.002% thiamine pyrophosphate, 15mM MgCl_2_, 5.3mM donor, and 16mM acceptor. After 24h of the catalysing reaction, sugar phosphates in the product were dephosphorylated using acid phosphatase. The dephosphorylated products were purified on a column containing ion-exchanging bed resin AG 501-X8(D) (Bio-Rad). The flow-through fractions were used for GC/MS analysis. (C) The chromatogram of the TMS derivatives of the dephosphorylated reaction products with glucose-6-phosphate as acceptor substrate and fructose-6-phosphate as donor (upper part) and mass spectra of the TMS derivative of octulose (lower part). The peak corresponding to the internal standard xylitol is labelled by a triangle and that of octulose is labelled by an asterisk. The reaction conditions are the same as described in (B). (D) The chromatogram of the TMS derivatives of the dephosphorylated reaction products with ribose-5-phosphate as acceptor substrate and β-hydroxypyruvate as donor (upper part) and the mass spectra of the TMS derivative of sedoheptulose (lower part). The peak corresponding to the internal standard xylitol is labelled by a triangle and that of sedoheptulose is labelled by an asterisk. The reaction conditions are the same as described in (B). (E) The chromatogram of the TMS derivatives of the dephosphorylated reaction products with ribose-5-phosphate as acceptor substrate and fructose-6-phosphate as donor (upper part) and the mass spectra of the TMS derivative of sedoheptulose (lower part). The peak corresponding to the internal standard xylitol is labelled by a triangle and that of sedoheptulose is labelled by an asterisk. The reaction conditions are the same as described in (B).

Protein extracts enriched in transketolase were obtained from leaves of *C. plantagineum* by fractionated ammonium sulphate precipitation. Immunoblotting showed that transketolase was concentrated in the fractions precipitated by 50–70% (w/v) ammonium sulphate (Fig. 2). This transketolase containing protein fraction was used in reactions with glucose-6-phosphate and β-hydroxypyruvate as substrates. Octulose was detected in the reaction by verifying that its trimethylsilyl derivative has the same mass spectrum as that of the octulose standard purified from *C. plantagineum* leaves (Fig. 3A, B). Octulose-8-phosphate was synthesized in the reaction which confirmed a previous result that octulose-8-phosphate was synthesized (Willige *et al.*, 2009). Octulose-8-phosphate was also synthesized when β-hydroxypyruvate was substituted by fructose-6-phosphate (Fig. 3C). However, when ribose-5-phosphate was the acceptor substrate in the reaction, sedoheptulose-7-phosphate was formed, despite β-hydroxypyruvate or fructose-6-phosphate being the donor (Fig. 3 D, E).

The genes encoding transketolases 3, 7, and 10 were cloned in the expression vector pET28a+ and the corresponding recombinant proteins were expressed and purified from isopropyl β-d-1-thiogalactopyranoside-induced *E. coli* (BL21 DE3) cells. The quality and identity of the proteins was validated by SDS-PAGE (see Supplementary Fig. S1 at *JXB* online). Various reactions were carried out with the purified recombinant proteins and the dephosphorylated products of the reactions were analysed by GC/MS (Table 1). Results showed that transketolase 7 and transketolase 10 of *C. plantagineum* had the ability to catalyse the formation of octulose-8-phosphate using glucose-6-phosphate as acceptor and β-hydroxypyruvate or fructose-6-phosphate as donor, whereas transketolase 3 of *C. plantagineum* could not perform this function. However, all three recombinant proteins catalysed the formation of sedoheptulose-7-phosphate with ribose-5-phosphate as acceptor substrate and β-hydroxypyruvate or fructose-6-phosphate as donor. Neither octulose nor sedoheptulose were detected in the dephosphorylated products of reactions catalysed by the proteins purified from *E. coli* (BL21 DE3) cells transformed with the vector pET28a+.

**Table 1.** Summary of the dephosphorylated products obtained in transketolase-catalysed reactions in combination with different acceptor and donor substrates

Enzyme^*a*^	Donor+acceptor	Donor+acceptor	Donor+acceptor	Donor+acceptor
	HP+Glu-6-P^*b*^	HP+Rib-5-P^*b*^	Fru-6-P+Glu-6-P^*b*^	Fru-6-P+Rib-5-P^*b*^
CK	–	–	–	–
CKe	–	–	–	–
Tkt3	–	Sed^*c*^	–	Sed
Tkt7	Oct^*c*^	Sed	Oct	Sed
Tkt10	Oct	Sed	Oct	Sed
Tktp	Oct	Sed	Oct	Sed

^*a*^ CK: reaction without protein; Cke: proteins purified from *E. coli* (BL21) transformed with vector pET28a+; Tktp: the protein enriched from leaf extracts of *C. plantagineum*.

^*b*^ HP refers to β-hydroxypyruvate, Glu-6-P to glucose-6-phosphate, Rib-5-P to ribose-5-phosphate, and Fru-6-P to fructose-6-phosphate.

^*c*^ Sed: sedoheptulose; Oct: octulose; –: no sedoheptulose or octulose found.

These results indicate that the exchange reaction is feasible with a transketolase-enriched protein fraction from *C. plantagineum*. The recombinant *C. plantagineum* transketolases 7 and 10 also demonstrate the feasibility of the exchange reaction (Table 1). The differentiation of the catalysing functions of the three transketolase isoforms demonstrates that they have different catalytic properties which are connected with different roles in sugar metabolism in *C. plantagineum*.

### Localization and transport of octulose

**Fig. 4.**
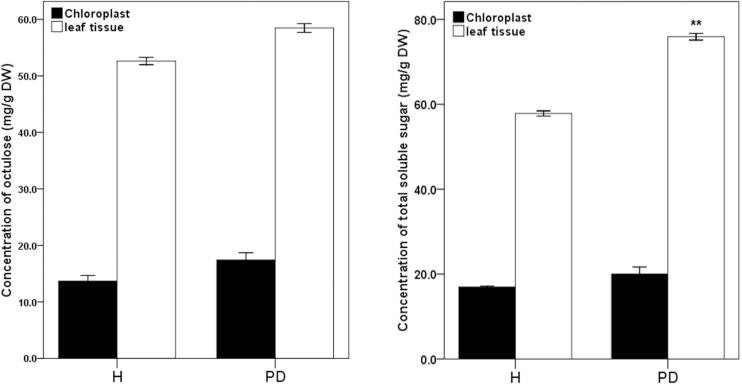
The amounts of sugars in chloroplasts and leaf tissues in hydrated (H) and partially dehydrated (PD) conditions. In PD, plants were slowly dehydrated for 2 d when an RWC of 75% was reached in the leaves. All data represent means ±SD (*n*=3). Asterisks indicate significant differences determined with Student’s *t* test (*P <0.05, **P <0.01).

Although octulose accumulates to a high level, its cellular localization is as yet unknown. Therefore we attempted to identify the cellular compartment where octulose accumulates and how it is transported. Experimental results showed that the amount of octulose in isolated chloroplasts was lower than in whole leaf tissues in hydrated conditions or partially dehydrated conditions (Fig. 4). Partial dehydration led to a significant increase in total soluble sugars in leaf tissue tissues but it did not affect the levels of octulose and total soluble sugars in the chloroplast. This implies that octulose mainly accumulates in the cytosol.

**Fig. 5.**
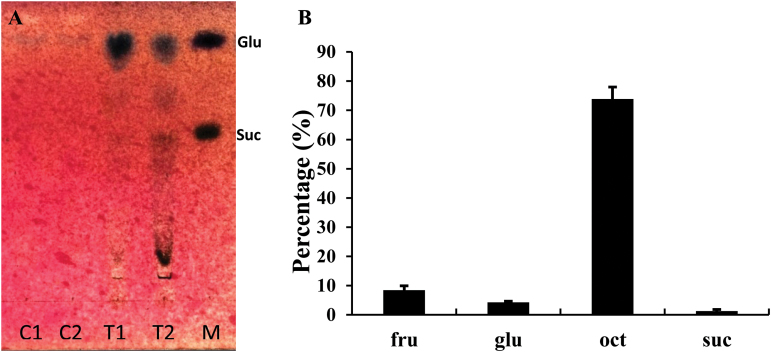
Thin layer chromatogram (a) and GC analysis of leaf exudates (b) of *C. plantagineum*. C1 and C2 are two replicates of the sugars exported into 5mM CaCl_2_ from leaves (control); T1 and T2 are two replicates of the sugars exported into 15mM EDTA (leaf exudates); Lane M contains glucose (Glu) and sucrose (Suc) standards. The GC analysis shows the percentage of sugars relative to total sugars. All data represent means ±SD (*n*=3).

Octulose was found to be the most abundant sugar in fully hydrated plants. However, a dedicated analysis showed that octulose levels fluctuate and are subject to a circadian rhythm (Supplementary Fig. S2). To explore the circadian change in octulose levels, phloem exudate analysis was conducted which demonstrated that 75–80% of the exported sugars is octulose (Fig. 5).

## Discussion

As an important component of the photosynthesis reaction and the pentose phosphate pathway, the evolution of transketolase in plants might reflect the strategy of plants in adapting sugar metabolism to environmental requirements. Phylogeny analysis of transketolase genes demonstrates that TKT7 and TKT10 in *C. plantagineum* have diverged from the transketolase genes of many desiccation-sensitive species. The sequence analysis showed that *C. plantagineum* TKT7 and TKT10 and their homologous genes (e.g. con 8 lb from *L. brevidens* and con 3 ls from *L. subracemosa*) lack recognizable transit peptides that transport the transketolase to the chloroplast. The transit peptide targeting transketolase to plastids is characteristic for most transketolase genes and is also present in TKT3 of *C. plantagineum* (Lange *et al.* 1998; Willige *et al.*, 2009). This may explain the diversification of *C. plantagineum* TKT7 and TKT10 and their homologous genes from *L. brevidens* and *L. subracemosa* and may be related to the fact that they encode enzymes involved in octulose metabolism in these plants.

Our results showed that TKT3 catalysed the transfer of a two-carbon ketol group from β-hydroxypyruvate or fructose-6-phosphate to ribose-5-phosphate to form sedoheptulose-7-phosphate. Besides catalysing the same transfer reaction, TKT7 and TKT10 also catalyse the formation of octulose-8-phosphate using glucose-6-phosphate as acceptor and β-hydroxypyruvate/fructose-6-phosphate as donor. This means that the exchange reaction is possible in *C. plantagineum* as it is in spinach (Williams and MacLeod, 2006). Willige *et al.* (2009) reported that TKT3 is localized in the chloroplasts while TKT7 and TKT10 are localized in the cytoplasm. Combined with the alternative Calvin cycle (Flanigan *et al.*, 2006), it is reasonable to suggest that TKT3 is only involved in carbon reactions in photosynthesis and the pentose phosphate pathway, while TKT7 and TKT10 are responsible for the accumulation of octulose in *C. plantagineum*. TKT7 and TKT10 could also participate in the pentose phosphate pathway, as transketolases can accept various substrates (Williams *et al.*, 1987; Krüger and von Schaewen, 2003) which is also shown for both TKT7 and TKT10 in our study. Expression analysis of transketolase isoforms showed that *TKT3* is constitutively expressed in leaves and roots while *TKT7* and *TKT10* are up-regulated in leaves during rehydration of the desiccated plant (Bernacchia *et al.*, 1995). The up-regulation of *TKT7* and *TKT10* positively correlates with octulose accumulation during rehydration. This supports the notion that TKT7 and TKT10 catalyse the exchange reaction to produce octulose. However, we are still left with the question: Is the conversion from octulose to sucrose during rehydration achieved through the reverse exchange reaction or is there another pathway for the conversion? Although the transcript levels of TKT7 and TKT10 decrease during dehydration (Rodriguez *et al.*, 2010), dehydration treatments generally take more than 2 weeks compared with rehydration that is completed within 48h. It is possible to achieve the conversion during the longer time period. It is also possible that octulose is converted to sucrose by another pathway that might comprise phosphotransferase, aldolase, triosephosphate isomerase, and fructose 1,6-*bis*phosphatase as proposed by Williams and MacLeod (2006). Further experimentation is necessary to verify this hypothesis.

Except in *C. plantagineum*, octulose was also found to accumulate in several closely related Linderniaceae, such as *Lindernia brevidens*, *Lindernia subracemosa*, *Lindernia philcoxii*, *Lindernia numilarifolia*, *Lindernia exilis*, and *Lindernia acicularis* (Kutzer, 2004; Phillips *et al.*, 2008). Thus it can be speculated that the exchange reaction catalysed by transketolase is also present in these plant species. Similarly, the other isomers of octulose, such as d-glycero-dmanno-octulose isolated from avocado and sedum species (Charlson and Richtmyer, 1960) and d-glycero-l-galacto-octulose isolated from *Persea gratissima*, *Sedum spectabile*, and *Primula oficinalis* (Sephton and Richtmyer, 1963; Begbie and Richtmyer, 1966), may also be synthesized by the exchange reactions catalysed by transketolase with substrates of specific stereo configurations.

Many monosaccharides do not occur naturally in the free state but are commonly found as phosphate-ester derivatives that are important intermediates in the breakdown and synthesis of carbohydrates in living organisms (Robyt, 1998). For some free monosaccharides, specific phosphatases exist, catalysing the dephosphorylation of their phosphate-ester derivatives, e. g. glucose and sedoheptulose (Van Schaftingen and Gerin, 2002; Ceusters *et al.*, 2013). It is likely that a phosphatase exists which dephosphorylates octulose-8-phosphate or its isomers to produce octulose in *C. plantagineum* or d-glycero-d-manno-octulose/d-glycero-l-galacto-octulose in *Persea gratissima*/*Sedum spectabile.*

Octulose was shown to be the dominant sugar in leaf exudates by phloem exudate analysis. This indicates that *C. plantagineum* leaves transport octulose to roots as an energy supply. Although, in general, sucrose is the main transport form in plants, some plants also transport raffinose and stachyose and/or sugar alcohols (Turgeon and Wolf, 2009). Some studies have also proposed sedoheptulose as the sugar transport form (Liu *et al.*, 2002; Ceusters *et al.*, 2013) and, therefore, octulose is a good candidate for sugar transport in *C. plantagineum* and related plants. Our study showed that octulose accumulation follows a circadian rhythm (Supplementary Fig. S2) as already indicated by Norwood *et al.* (2003). The circadian fluctuation in octulose levels might be explained by octulose transport or by the circadian regulation of biosynthetic enzyme activities.

Sugars are often seen as an energy resource and as signalling molecules in plant cells (Eveland and Jackson, 2012). In the present study, we compared octulose levels in isolated chloroplasts and intact leaf tissues which suggests that octulose is localized in the cytosol. It is also possible that octulose is stored in the vacuole which occupies >95% of the cell volume in fully turgid plants and could serve as sites for storing soluble carbohydrates. This hypothesis could not be proved, as a protocol for vacuole isolation for *C. plantagineum* could not yet be established. Additional experiments showed that partial dehydration did not affect octulose levels in the leaf tissues. This can be explained by the study of Kutzer (2004) which showed that, during dehydration, sucrose starts to be rapidly synthesized in *C. plantagineum* only when the RWC is below 75%. Similarly, Cooper and Farrant (2002) found that sucrose substantially accumulated in *Craterostigma wilmsii* only during the late stages of dehydration (below 25% RWC). Increasing evidence also suggests that sugar molecules counteract oxidative stress by acting as genuine ROS scavengers (Van den Ende and Valluru, 2009; Peshev *et al.*, 2013). Our analysis with the octulose isolated from TLC plates suggested that octulose has a stronger hydroxyl radical (OH^−^) scavenging ability than sucrose (Zhang and Bartels, 2016). Previous studies showed that sucrose is a more efficient scavenger than glucose and fructose (Nishizawa *et al.*, 2008; Peshev *et al.*, 2013). The superior antioxidant properties of octulose might also be a reason for the high accumulation in *C. plantagineum*.

**Fig. 6.**
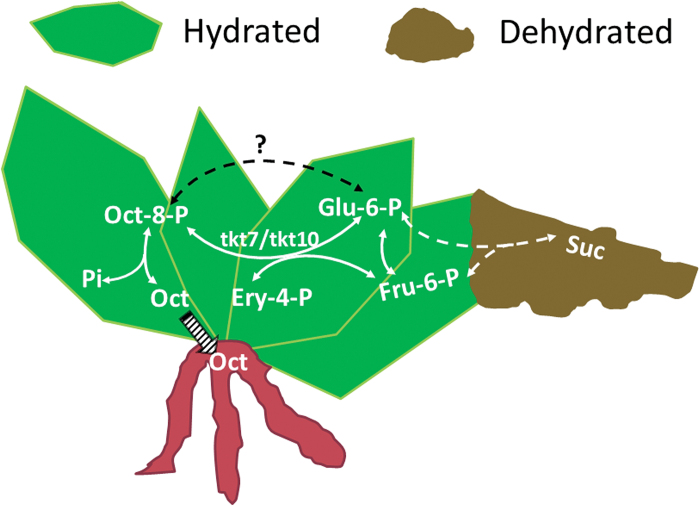
A scheme of octulose metabolism in *C. plantagineum*. In hydrated conditions, octulose-8-phosphate (Oct-8-P) is synthesized through the exchange reaction that is catalysed by transketolase 7 (tkt7) and transketolase 10 (tkt10) using glucose-6-phosphate (Glu-6-P) and fructose-6-phosphate (Fru-6-P) as substrates. Oct-8-P is dephosphorylated to produce octulose (Oct). Oct can be transported from leaves to roots. In dehydrated conditions, Oct-8-P is transformed into Glu-6-P or Fru-6-P that is used to synthesize sucrose (Suc). Pi refers to phosphate. The striped arrow indicates the transport of octulose from leaves to roots. The dashed arrow only indicates the overall reaction direction (the details of the reaction are not shown).

In conclusion, our study suggests that the *TKT7* and *TKT10* isoforms of transketolase show distinct specificity in function and evolution. They catalyse the synthesis of octulose-8-phosphate using glucose-6-phosphate and fructose-6-phosphate as substrate (Fig. 6). Octulose acts as a transport sugar in *C. plantagineum* and could play a role in antioxidant defence in the vacuole because of its stronger OH^−^ scavenging ability than other abundant sugars. To date, a scheme of the metabolism of octulose in *C. plantagineum* can be formed to explain the conversion between octulose and sucrose based on the cellular water status.

## Supplementary data

Supplementary data can be found at *JXB* online.

**Figure S1.** SDS-PAGE of purified proteins: transketolase 3 (a), transketolase 7 (b), and transketolase 10 (c).

**Figure S2.** Diurnal variation of octulose levels in leaves of *C. plantagineum*.

**Table S1.** List of primers used in this study.

Supplementary Data

## References

[CIT0001] Bartels D Engelhardt K Roncarati R Schneider K Rotter M Salamini F 1991 An ABA and GA modulated gene expressed in the barley embryo encodes an aldose reductase related protein. The EMBO Journal 10, 1037. 1827067 10.1002/j.1460-2075.1991.tb08042.x PMC452754

[CIT0002] Bartels D Schneider K Terstappen G Piatkowski D Salamini F 1990 Molecular cloning of abscisic acid-modulated genes which are induced during desiccation of the resurrection plant *Craterostigma plantagineum* . Planta 181, 27–34. 24196671 10.1007/BF00202321

[CIT0003] Begbie R Richtmyer NK 1966 The isolation of some heptoses, heptuloses, octuloses, and nonuloses from *Primula officinalis* Jacq. Carbohydrate Research 2, 272–288.

[CIT0004] Bernacchia G Schwall G Lottspeich F Salamini F Bartels D 1995 The transketolase gene family of the resurrection plant *Craterostigma plantagineum*: differential expression during the rehydration phase. The EMBO Journal 14, 610–618. 7859749 10.1002/j.1460-2075.1995.tb07037.x PMC398120

[CIT0005] Bianchi G Gamba A Murelli C Salamini F Bartels D 1991 Novel carbohydrate metabolism in the resurrection plant *Craterostigma plantagineum* . The Plant Journal 1, 355–359. 10.1046/j.1365-313X.1991.t01-11-00999.x 29345773

[CIT0006] Bradford MM 1976 A rapid and sensitive method for the quantitation of microgram quantities of protein utilizing the principle of protein–dye binding. Analytical Biochemistry 72, 248–254. 942051 10.1016/0003-2697(76)90527-3

[CIT0007] Ceusters J Godts C Peshev D Vergauwen R Dyubankova N Lescrinier E De Proft MP Van den Ende W 2013 Sedoheptulose accumulation under CO_2_ enrichment in leaves of *Kalanchoë pinnata*: a novel mechanism to enhance C and P homeostasis? Journal of Experimental Botany 64, 1497–1507. 23378377 10.1093/jxb/ert010 PMC3617823

[CIT0008] Charlson AJ Richtmyer NK 1960 The isolation of an octulose and an octitol from natural sources:d-glycero-d-manno-octulose anddD-erythro-d-galacto-octitol from the avocado andd-glycero-d-manno-octulose from *Sedum* species. Journal of the American Chemical Society 82, 3428–3434.

[CIT0009] Clasquin MF Melamud E Singer A et al 2011 Riboneogenesis in yeast. Cell 145, 969–980. 21663798 10.1016/j.cell.2011.05.022 PMC3163394

[CIT0010] Cooper K Farrant JM 2002 Recovery of the resurrection plant *Craterostigma wilmsii* from desiccation: protection versus repair. Journal of Experimental Botany 53, 1805–1813. 12147731 10.1093/jxb/erf028

[CIT0011] Dinakar C Bartels D 2013 Desiccation tolerance in resurrection plants: new insights from transcriptome, proteome, and metabolome analysis. Frontiers in Plant Science 4, 482. 24348488 10.3389/fpls.2013.00482 PMC3842845

[CIT0012] Eveland AL Jackson DP 2012 Sugars, signalling, and plant development. Journal of Experimental Botany 63, 3367–3377. 22140246 10.1093/jxb/err379

[CIT0013] Flanigan IL MacLeod JK Williams JF 2006 A re-investigation of the path of carbon in photosynthesis utilizing GC/MS methodology. Unequivocal verification of the participation of octulose phosphates in the pathway. Photosynthesis Research 90, 149–159. 17149533 10.1007/s11120-006-9114-4 PMC1779625

[CIT0014] Hall BG 2013 Building phylogenetic trees from molecular data with MEGA. Molecular Biology and Evolution 30, 1229–1235. 23486614 10.1093/molbev/mst012

[CIT0015] Hoekstra FA Wolkers WF Buitink J Golovina EA Crowe JH Crowe LM 1997 Membrane stabilization in the dry state. Comparative Biochemistry and Physiology Part A: Physiology 117, 335–341.

[CIT0016] Ingram J Chandler JW Gallagher L Salamini F Bartels D 1997 Analysis of cDNA clones encoding sucrose-phosphate synthase in relation to sugar interconversions associated with dehydration in the resurrection plant *Craterostigma plantagineum* Hochst. Plant Physiology 115, 113–121. 9306694 10.1104/pp.115.1.113 PMC158466

[CIT0017] Kirch HH Röhrig H 2010 Affinity purification and determination of enzymatic activity of recombinantly expressed aldehyde dehydrogenases. Methods in Molecular Biology 639, 282–291. 20387053 10.1007/978-1-60761-702-0_17

[CIT0018] Kleines M Elster R-C Rodrigo M-J Blervacq A-S Salamini F Bartels D 1999 Isolation and expression analysis of two stress-responsive sucrose-synthase genes from the resurrection plant *Craterostigma plantagineum* (Hochst.). Planta 209, 13–24. 10467027 10.1007/s004250050602

[CIT0019] Krüger NJ von Schaewen A 2003 The oxidative pentose phosphate pathway: structure and organisation. Current Opinion in Plant Biology 6, 236–246. 12753973 10.1016/s1369-5266(03)00039-6

[CIT0020] Kutzer M 2004 Untersuchung zum Zuckerstoffwechsel der Wiederauferstehungspflanze *Craterostigma plantagineum* und einiger Lindernia-Arten. PhD thesis, University of Bonn, Germany.

[CIT0021] Laemmli U 1970 Most commonly used discontinuous buffer system for SDS electrophoresis. Nature 227, 680–685.5432063

[CIT0022] Lange MB Wildung MR McCaskill D Croteau R 1998 A family of transketolases that directs isoprenoid biosynthesis via a mevalonate-independent pathway. Proceedings of the National Academy of Sciences, USA 95, 2100–2104. 10.1073/pnas.95.5.2100 PMC19263 9482845

[CIT0023] Liu X Sievert J Arpaia ML Madore MA 2002 Postulated physiological roles of the seven-carbon sugars, mannoheptulose, and perseitol in avocado. Journal of the American Society for Horticultural Science 127, 108–114.

[CIT0024] Nishizawa A Yabuta Y Shigeoka S 2008 Galactinol and raffinose constitute a novel function to protect plants from oxidative damage. Plant Physiology 147, 1251–1263. 18502973 10.1104/pp.108.122465 PMC2442551

[CIT0025] Norwood M Toldi O Richter A Scott P 2003 Investigation into the ability of roots of the poikilohydric plant *Craterostigma plantagineum* to survive dehydration stress. Journal of Experimental Botany 54, 2313–2321. 12947051 10.1093/jxb/erg255

[CIT0026] Peshev D Vergauwen R Moglia A Hideg E Van den Ende W 2013 Towards understanding vacuolar antioxidant mechanisms: a role for fructans? Journal of Experimental Botany 64, 1025–1038. 23349141 10.1093/jxb/ers377 PMC3580814

[CIT0027] Peters S Mundree SG Thomson JA Farrant JM Keller F 2007 Protection mechanisms in the resurrection plant *Xerophyta viscosa* (Baker): both sucrose and raffinose family oligosaccharides (RFOs) accumulate in leaves in response to water deficit. Journal of Experimental Botany 58, 1947–1956. 17452754 10.1093/jxb/erm056

[CIT0028] Phillips JR Fischer E Baron M Van Den Dries N Facchinelli F Kutzer M Rahmanzadeh R Remus D Bartels D 2008 *Lindernia brevidens*: a novel desiccation-tolerant vascular plant, endemic to ancient tropical rainforests. The Plant Journal 54, 938–948. 18346195 10.1111/j.1365-313X.2008.03478.x

[CIT0029] Robyt JF 1998 *Essentials of carbohydrate chemistry*. Springer Science & Business Media.

[CIT0030] Rodriguez MCS Edsgärd D Hussain SS Alquezar D Rasmussen M Gilbert T Nielsen BH Bartels D Mundy J 2010 Transcriptomes of the desiccation-tolerant resurrection plant *Craterostigma plantagineum* . The Plant Journal 63, 212–228. 20444235 10.1111/j.1365-313X.2010.04243.x

[CIT0031] Rowan B Bendich A 2011 Isolation, quantification, and analysis of chloroplast DNA. In: Jarvis RP, ed. *Chloroplast research in Arabidopsis*: *methods and protocols*, Vol. 1 Humana Press: New York, 151–170. 10.1007/978-1-61779-234-2_10 21822838

[CIT0032] Sambrook J Russell DW Irwin N 2001 *Molecular cloning: a laboratory manual*. Cold Spring Harbor Laboratory Press: Cold Spring Harbor, NY.

[CIT0033] Sephton HH Richtmyer NK 1963 The isolation of a second octulose and of a heptose from the Avocado:d-glycero-l-galacto-octulose andd-glycero-d-galacto-heptose. The Journal of Organic Chemistry 28, 1691–1694.

[CIT0034] Turgeon R Wolf S 2009 Phloem transport: cellular pathways and molecular trafficking. Annual Revivew of Plant Biology 60, 207–221. 10.1146/annurev.arplant.043008.092045 19025382

[CIT0035] Van den Ende W Valluru R 2009 Sucrose, sucrosyl oligosaccharides, and oxidative stress: scavenging and salvaging? Journal of Experimental Botany 60, 9–18. 19036839 10.1093/jxb/ern297

[CIT0036] Van Schaftingen E Gerin I 2002 The glucose-6-phosphatase system. Biochemical Journal 362, 513–532. 11879177 10.1042/0264-6021:3620513 PMC1222414

[CIT0037] Vicré M Farrant J Driouich A 2004 Insights into the cellular mechanisms of desiccation tolerance among angiosperm resurrection plant species. Plant, Cell and Environment 27, 1329–1340.

[CIT0038] Williams JF Arora KK Longenecker JP 1987 The pentose pathway: a random harvest: impediments which oppose acceptance of the classical (F-type) pentose cycle for liver, some neoplasms and photosynthetic tissue. The case for the L-type pentose pathway. International Journal of Biochemistry 19, 749–817. 3319734 10.1016/0020-711x(87)90239-4

[CIT0039] Williams JF MacLeod JK 2006 The metabolic significance of octulose phosphates in the photosynthetic carbon reduction cycle in spinach. Photosynthesis Research 90, 125–148. 17160443 10.1007/s11120-006-9113-5 PMC1779624

[CIT0040] Willige BC Kutzer M Tebartz F Bartels D 2009 Subcellular localization and enzymatic properties of differentially expressed transketolase genes isolated from the desiccation tolerant resurrection plant *Craterostigma plantagineum* . Planta 229, 659–666. 19052774 10.1007/s00425-008-0863-5

[CIT0041] Wingenter K Schulz A Wormit A Wic S Trentmann O Hoermiller II Heyer AG Marten I Hedrich R Neuhaus HE 2010 Increased activity of the vacuolar monosaccharide transporter TMT1 alters cellular sugar partitioning, sugar signaling, and seed yield in Arabidopsis. Plant Physiology 154, 665–677. 20709831 10.1104/pp.110.162040 PMC2949046

[CIT0042] Zhang Q Bartels D 2016 Physiological factors determine the accumulation ofd-glycero-d-ido-octulose (d-g-d-i-oct) in the desiccation tolerant resurrection plant *Craterostigma plantagineum* . Functional Plant Biology (in press). 10.1071/FP15278 32480496

