# Supplementary material for: The role of transketolase and octulose in the resurrection plant Craterostigma plantagineum
Source: J Exp Bot. 2016 Apr 29;67(11):3551–9. doi: 10.1093/jxb/erw174 (PMC4892735; doi:10.1093/jxb/erw174)
Supplement: Supplementary Data [file supp_67_11_3551__index.html]

The role of transketolase and octulose in the resurrection plant Craterostigma plantagineum — The role of transketolase and octulose in the resurrection plant Craterostigma plantagineum — Supplementary Data 

# The role of transketolase and octulose in the resurrection plant *Craterostigma plantagineum*

## Supplementary Data

Data files

- supplementary\_figures\_S1\_S2\_table\_S1.pdf - Supplementary Data
