# Supplementary material for: The role of transketolase and octulose in the resurrection plant Craterostigma plantagineum
Source: J Exp Bot. 2016 Apr 29;67(11):3551–9. doi: 10.1093/jxb/erw174 (PMC4892735; doi:10.1093/jxb/erw174)
Supplement: Supplementary Data [file supp_erw174_supplementary_figures_S1_S2_table_S1.pdf]

Supplementary data

The role of transketolase and octulose in the resurrection plant *Craterostigma plantagineum*

Qingwei Zhang, Thomas Vitus Linnemann, Lukas Schreiber, Dorothea Bartels*

*Author for correspondence; e-mail: dbartels@uni-bonn.de;

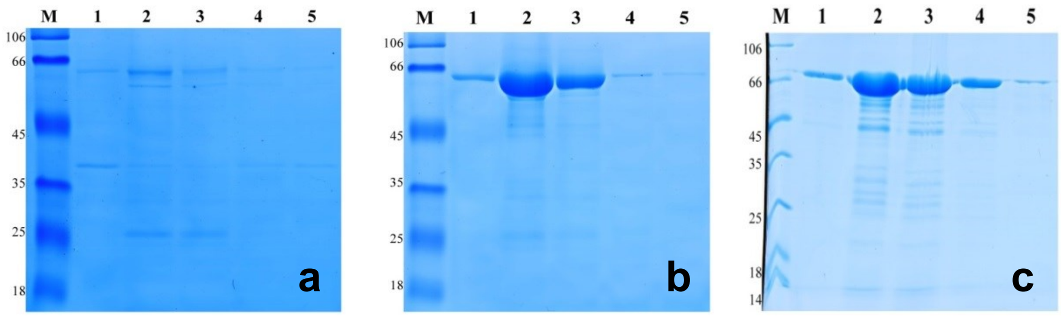

Fig. S1. SDS-PAGE of purified proteins: transketolase 3 (a), transketolase 7 (b) and transketolase 10 (c). From left to right in each sub-figure lanes represent the protein size markers (M) and the eluted fractions 1 to 5. The protein size markers are given in kDa.

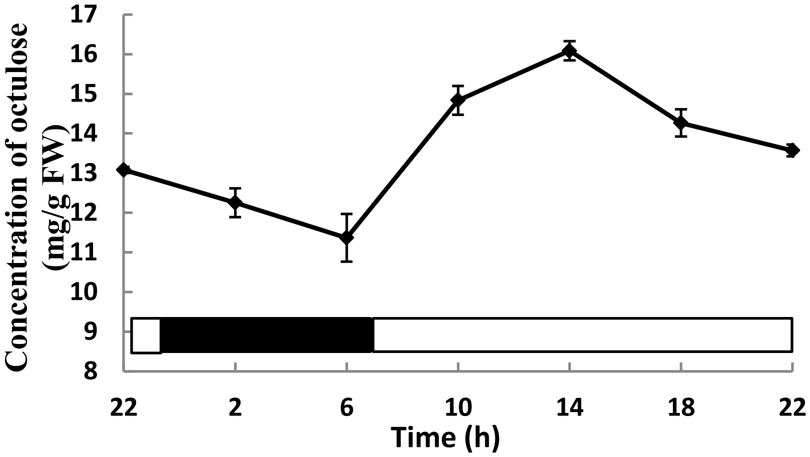

Fig. S2. Concentrations of octulose in *C. plantagineum* leaves within a 24-h period. Samples were collected each four hours and first sampling began at 22:00. The black bar represents the dark period and the transparent bar the light period. All data represent means ± SD (n=3).

Table S1 Sequences of primers used in this study.

Primer name	Sequence 5'-3'
tk3-Forward	CGAGGATCCATGGAAGGGGTTTCTAACGAG
tk3-Reverse	GCCAAGCTTTCAAATCAACTCCTTCGCAGC
t7-Forward	ATAGAATTCATGGCGCCCAAGACG
t7-Reverse	CCGAAGCTTTCAGCAAATCTCCTT
t10-Forward	TAAGGATCCATGGCCAAGACTACG
t10-Reverse	CCTAAGCTTCTAGCACAGCTCTTT

The sequences underlined are restriction enzyme sites.
